# Animal Models of Bone Loss in Inflammatory Arthritis: from Cytokines in the Bench to Novel Treatments for Bone Loss in the Bedside—a Comprehensive Review

**DOI:** 10.1007/s12016-015-8522-7

**Published:** 2015-12-03

**Authors:** C. Henrique Alves, Eric Farrell, Marijn Vis, Edgar M. Colin, Erik Lubberts

**Affiliations:** 1 Department of Rheumatology, Erasmus MC, University Medical Center, Wytemaweg 80, P.O. Box 2040, 3000 CA Rotterdam, The Netherlands; 2 Department of Immunology, Erasmus MC, University Medical Center, Rotterdam, P.O. Box 2040, 3000 CA Rotterdam, The Netherlands; 3 Department of Oral and Maxillofacial Surgery, Special Dental Care and Orthodontics, Erasmus MC, University Medical Center, Rotterdam, P.O. Box 2040, 3000 CA Rotterdam, The Netherlands; 4 Department of Rheumatology, ZGT Almelo, Zilvermeeuw 1, 7600 SZ Almelo, The Netherlands

**Keywords:** Arthritis, Inflammation, Osteoclast, Osteoblast, Cytokine, IL-17

## Abstract

Throughout life, bone is continuously remodelled. Bone is formed by osteoblasts, from mesenchymal origin, while osteoclasts induce bone resorption. This process is tightly regulated. During inflammation, several growth factors and cytokines are increased inducing osteoclast differentiation and activation, and chronic inflammation is a condition that initiates systemic bone loss. Rheumatoid arthritis (RA) is a chronic inflammatory auto-immune disease that is characterised by active synovitis and is associated with early peri-articular bone loss. Peri-articular bone loss precedes focal bone erosions, which may progress to bone destruction and disability. The incidence of generalised osteoporosis is associated with the severity of arthritis in RA and increased osteoporotic vertebral and hip fracture risk. In this review, we will give an overview of different animal models of inflammatory arthritis related to RA with focus on bone erosion and involvement of pro-inflammatory cytokines. In addition, a humanised endochondral ossification model will be discussed, which can be used in a translational approach to answer osteoimmunological questions.

## Bone Destruction in Rheumatoid Arthritis

### Osteoclast Differentiation in Inflammatory Arthritis

Osteoclasts are multinucleated motile cells derived from hematopoietic stem cells during a multistep differentiation process called osteoclastogenesis through cell-cell contact or the secretion of local factors. Osteoclast differentiation is regulated by two essential cytokines: the macrophage colony-stimulating factor-1 (M-CSF) and the receptor activator of nuclear factor-kB ligand (RANKL) [1, 2]. M-CSF is considered a crucial factor responsible for the survival and proliferation of osteoclast precursor cells [3]. RANKL was cloned from activated T cells as a new member of the tumour necrosis factor (TNF) superfamily in 1997 [4]. RANKL is expressed by cells of mesenchymal origin in bone and is a direct regulator of osteoclast formation and bone turnover. In addition, cells of the immune system, such as T cells [5, 6] and B cells express RANKL [2, 7, 8]. The role of RANKL as the critical osteoclastogenic factor was demonstrated using knockout mice. These mice developed severe osteopetrosis due to complete absence of osteoclasts [9]. The receptor for RANKL is RANK, a type I transmembrane protein expressed on osteoclast precursor cells and mature osteoclasts. The binding of RANKL to RANK is inhibited by the decoy receptor osteoprotegerin (OPG). The RANKL/OPG ratio is an important determinant for the balance in osteoclastogenesis.

Osteoclasts are designed to resorb bone by generating a sealing zone, an actin ring made of densely packed podosomes, beneath their ruffled border, into which they secrete hydrochloric acid to solubilise calcium from bone and proteolytic enzymes, such as matrix metalloproteinases, cathepsin K and phosphatases (tartrate-resistant acid phosphatase (TRAP)) to degrade the remaining matrix.

Increase of osteoclast numbers and activity is a hallmark of inflammatory bone loss. The inflammatory microenvironment promotes not only precursor recruitment from bone marrow but also their subsequent differentiation into mature osteoclasts. The first indirect description of bone-resorbing cells in RA dates back to the nineteenth century [10] and was revised more than a century later by Bromley and Woolley [11] and Leisen [12]. Osteoclast in RA were recently identified and characterised in more detail by Gravallese et al. [13]. It was also demonstrated that RA patients with active disease have greater synovial RANKL expression when compared with those with lower disease activity, and this increase in RANKL is accompanied by a decrease in OPG expression [14, 15], leading to a microenvironment that favours osteoclastogenesis. Osteoclast precursor cells accumulate inside the dense inflammatory synovially derived tissue located both at the interface with bone and inside the bone erosions themselves (synovial pannus).

Similar changes are also found in animal models of inflammatory arthritis, described in detail in ‘Critical Processes in Bone Remodelling in Animal Models of Arthritis’. Studies involving these demonstrated the crucial role of osteoclasts in the pathogenesis of articular bone erosion in arthritis induced by adjuvant [16], antigen [17], collagen [18–20], serum transfer [21–23] and TNF [24–26]. By inducing arthritis in osteoclast-free mice, it was shown that these mice are completely protected from bone erosions [21, 24]. Furthermore, RANKL knockout mice are also resistant to inflammation-induced bone loss [21].

### Osteoblast Differentiation in Inflammatory Arthritis

Derived from mesenchymal stem cells, osteoblast precursor cells require up-regulation of the Runx2 [27] and Osterix [28] transcription factors. Maturation to a non-proliferative matrix-producing osteoblast is marked by the expression of alkaline phosphatase, type 1 collagen along with non-collagen proteins such as osteocalcin, osteopontin and bone sialoprotein. Mature osteoblasts are capable of producing a characteristic extracellular collagenous matrix that subsequently becomes mineralised by deposition of hydroxyapatite crystals (reviewed in [29, 30]). Osteoblasts are not only responsible for the synthesis and mineralisation of bone, but they are also able to modulate osteoclast differentiation by stimulating osteoclast differentiation via production of M-CSF and RANKL [31, 32] or inhibiting osteoclast differentiation by OPG production [33, 34]. Other factors involved in osteoblast-osteoclast interaction include paracrine regulators, such as parathyroid hormone (PTH) and prostaglandin E2 (PGE2), which increase RANKL expression by osteoblasts [35, 36]. In addition, bone remodelling relies upon two other key pathways that regulate osteoblast differentiation and function, the canonical Wingless (Wnt) [37, 38] and the tumor growth factor-beta (TGF-β)/bone morphogenetic protein (BMP) [39] pathways.

As described in the previous section, osteoclasts are the principal cell type responsible for bone loss in RA [21, 40]; however, other cell types, including synovial fibroblasts and macrophages, might also contribute to bone erosion [41, 42]. Nevertheless, osteoblast differentiation and function has recently been suggested to be abnormal at sites of focal bone erosion in RA.

A murine study using the K/BxN model of serum transfer arthritis showed that the rate of bone formation is similar in arthritic and non-arthritic bone, suggesting that in RA, increased osteoclast resorption activity is not counterbalanced by osteoblast driven bone formation. Furthermore, within the arthritic bone, mineralisation of the newly formed bone in areas adjacent to sites of inflammation is reduced compared with bone surfaces adjacent to normal bone marrow. This suggests that inflammatory tissue impairs osteoblast activity [43]. Using the same K/BxN model of serum transfer arthritis, inflammation and bone erosion were induced and inflammation was then allowed to resolve completely. Proceeding inflammation resolution a significant increase in bone formation at previous inflammation-bone interfaces is observed, correlating with altered synovial expression of Wnt signalling components that favour anabolic signalling [44].

The Wnt signalling antagonist Dickkopf-1 (DKK-1) is increased in the mouse model overexpressing human TNF-α (hTNF.Tg). Prophylactic treatment with an antibody against DKK-1 prevented focal erosion, an effect also due in part to up-regulation of OPG expression in synovial tissue, suggesting that DKK-1 can act to inhibit bone formation [45]. In line with this, DKK-1 levels are higher in the sera of patients with RA than in either normal controls or patients with ankylosing spondylitis. Levels of DKK-1 correlate with disease activity in RA [46].

Other factors such as hypoxia and reduced pH in the arthritic bone microenvironment also play a role in impaired bone formation [47, 48].

Although uncommon, repair of bone erosions through formation of new bone has been described in patients undergoing conventional disease-modifying anti-rheumatic drug (DMARDs) therapy. Moreover, the repair occurred predominantly in patients with low disease activity at the time of follow-up [49].

## Critical Processes in Bone Remodelling in Animal Models of Arthritis

Disruption of the balance between bone resorption and formation is observed in several rheumatic diseases. Pro-inflammatory cytokines, as IL-1, IL-6, IL-17 and TNF-α, are able to stimulate osteoclast differentiation by expansion of the osteoclast precursor pool, up-regulation of RANKL expression in osteoblasts and/or in synovial fibroblasts and by synergism with RANKL itself, normally resulting in bone loss [26, 50, 51]. Pro-inflammatory cytokines, such as IL-1, IL-6 and TNF-α, can be found in high concentrations in the synovial fluid and tissues of RA patients. Chronic inflammation in RA leads to focal articular bone erosions within inflamed joints, as well as generalised osteoporosis. This bone loss progresses throughout the disease, correlates with disease severity and if untreated, it might progress to joint deformity and fractures [52]. Pro-inflammatory cytokines, secreted predominantly by macrophages, synovial fibroblasts and lymphocytes within the inflamed synovium and pannus, mediate the erosive process by enhancing osteoclast differentiation and activity.

Animal models provide the opportunity for investigating the pathogenesis of RA, to unravel the mechanism related with bone resorption/formation, to identify drug targets and test potential therapeutics. These models include induced and genetically manipulated arthritis models. In this section, we will describe some of the most commonly used arthritis models (Table 1) and discuss the role of critical cytokines in each model (Table 2).

**Table 1:** Characteristic of selected mouse models of arthritis

Arthritis model	Feature	Bone erosion		Irreversible cartilage surface erosion		Inflammation					Auto-antibodies		References
						Th cells	Cytokines			IC mediated			
Cartilage directed autoimmunity													
Collagen-induced arthritis (CIA)	CII autoimmune	+	+		+		+		+		CII	[53, 54]	
Infectious agents/exogenous triggers													
Streptococcal cell wall (SCW)	Acute	Peptidoglycan-polysaccharide	−		−		−		+		−	[55]	
	Reactivation		+		+/−		+		+		+/−		
SCW-flare	Memory T cells	+	+/−		+		+		−			[56]	
Antigen-induced arthritis (AIA)	Persistent antigen	+	+/−		+		+		+		−	[57]	
AIA	Persistent antigen	+	−		+		+		−		−	[58]	
AIA-flare	Memory T cells	+	+/−		+		+		−			[59]	
Transgenic spontaneous models													
K/BxN	GPI, T cell defect	+	+		+			+	+		GPI	[60]	
SKG	ZAP-70, T cell defect	+?	?		+			+	−			[61]	
Gp130	STAT3, T cell defect	+?	?		+			+	−			[62]	
IL-1Ra^−/−^	Autoimmune T cells	+	+		+			+	-		−	[63]	
hTNF.tg	TNF overexpression	+	+		−			+	−			[64]	
BXD2	T cell senescence	+	+		+			+	+			[65, 66]	
DNA II^−/−^IFN-IR^−/−^	DNA clearance	+	+		+?			+	+		CCP and RF	[67]	

*CII* collagen type II, *GPI* glucose-6-phosphatase isomerase, *IC* immune complexes, *CCP* cyclic citrullinated peptide, *RF* rheumatoid factor

**Table 2:** The contribution of pro-inflammatory cytokines to the arthritis development in selected mouse models of arthritis

Arthritis model	IL-17A	IL-6	IL-1	TNF	IL-23	References
CIA	+	+	++	+	+	[53, 54]
AIA	+	+	NR	+	+	[59]
SCW-acute	-	+	+	+	+	[68]
SCW-flare	+	+	+	+	+	[69]
K/BxN	+	+	+	+	NR	[70]
K/BxN serum transfer	-	-	++	+	+	[60]
SKG	++	++	+	+	NR	[61]
Gp130	NR	++	NR	NR	NR	[62]
IL-1Ra^-/-^	++	-	NR	++	NR	[63]
hTNF.tg	NR	-	++	++	NR	[64]
BXD2	++					[65, 66]
DNA II^−/−^IFN-IR^−/−^	+	+	+	+	NR	[67]

*NR* not reported

### Collagen-Induced Arthritis

The collagen-induced arthritis (CIA) model was first described in 1977, when Trentham and his colleagues reported that immunisation of rats with an emulsion of human, chick or rat type II collagen (CII) in complete Freund’s adjuvant (CFA) or incomplete Freund’s adjuvant (IFA) resulted in the development of an erosive polyarthritis associated with an auto-immune response against cartilage [53]. Others also described similar protocols for induction of CIA in mice [71] and in non-human primates [72]. One of the aspects of the immune response in this model is the production of CII-specific antibodies [73]. As in human RA, mice immunised with CII also produce rheumatoid factor [74]. The histology of CIA resembles RA and it is possible to observe cell infiltrate in synovial tissue and destruction of bone and cartilage (Table 1).

CIA susceptibility is linked to the expression of specific MHC class II molecules, which in mice is referred to as the H-2 complex. Strains expressing H-2^q^ or H-2^r^ are susceptible to CIA [75]. The induction of arthritis in mice of a C57BL/6 (H-2^b^) background [76] has facilitated the use of gene knockout mice and more recently by the generation of the congenic C57BL/6N.Q strain, which expresses the arthritis-susceptible q haplotype of the MHC class II region [77].

**Fig. 1:**
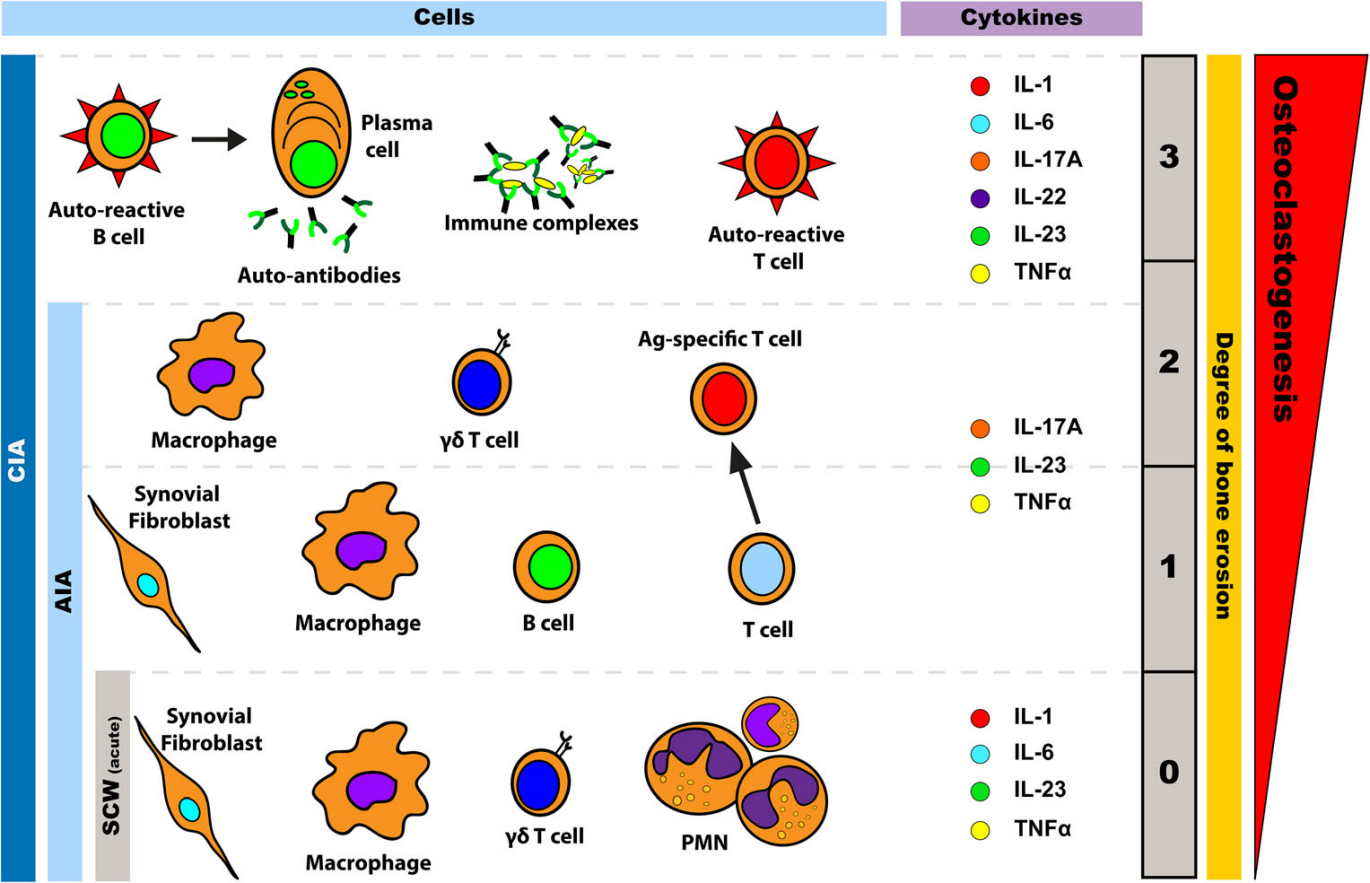
Cell types and cytokines involved in bone loss and arthritis development in different arthritis mouse models. The arthritis development in the streptococcal cell wall-induced arthritis (*SCW*) model is mediated by synovial fibroblast and innate immune cells as macrophages, γδ T cells and polymorphonuclear cells (*PMN*). These cells secrete IL-1, IL-6, IL-23 and TNF-α. No bone erosion is observed in this acute joint inflammation model. In the antigen-induced arthritis (*AIA*) model, macrophages, B cells and T cells are responsible for disease induction. In AIA, the main cytokines involved are IL-17A, IL-23 and TNF-α. In this model, mild (*1*) to moderate (*2*) bone erosion can be observed. The AIA flare-up model is driven mainly by antigen-specific memory T cells that activate synoviocytes leading to synovial inflammation within hours followed by joint destruction. The collagen-induced arthritis (*CIA*) is an erosive polyarthritis model associated with an auto-immune response against cartilage. CIA is mediated by auto-reactive T and B cells directed against type II collagen. B cells can be differentiated in plasma cells that produce auto-antibodies. Immune complex-mediated immune activation and complement play a role in the progression of the disease. In addition, many pro-inflammatory cytokines such as IL-1, IL-6, IL-17A, IL-22, IL-23 and TNF-α play a role in the development and/or progression of CIA. The degree of bone erosion can vary between mild (*1*) and severe (*3*)

The induction of CIA in mice is mediated by both auto-reactive T and B cells. Antigen-specific T cells are predominantly involved in the induction phase of the disease, supporting the activation of collagen-specific B cells and auto-antibodies. These pathogenic antibodies recognise their endogenous antigen in the joint resulting in complement activation, immune complex formation and triggering of a local inflammatory response including pro-inflammatory cytokines, whereby monocytes, granulocytes and T cells are attracted to the joint cavity (review in [78]) (Fig. 1; Table 2). Several studies demonstrated the importance of T cells in the induction of the disease in the CIA model. Holmdahl et al. showed that administration of CII-specific T cells can induce arthritis in naïve mice [79]. Moreover, mice deficient for CD4^+^ T cells are less susceptible to CIA than wild-type mice [80].

### Antigen-Induced Arthritis

Antigen-induced arthritis (AIA) is seen after intra-articular injection of protein antigen (e.g. methylated bovine serum albumin) into the knee joints of animals that have previously been immunised with the same antigen [81]. A swelling of the injected knee appears within the following days. The histopathological appearance of AIA bears similarities to RA, including synovial lining layer hyperplasia, perivascular infiltration with lymphocytes and plasma cells, lymphoid follicles, pannus and cartilage erosions. The erosiveness is related to the ability of the antigen to bind cartilage and is also dependent of the immunisation strategy used [58] (Fig. 1). However, unlike RA, AIA is a monoarticular disease that affects only the injected joints. When induced as described above, AIA is considered to be an acute disease model where the initial inflammation subsides after approximately 1–3 weeks, depending on the immunisation strategy [58]. Moreover, by locally re-injecting a small amount of antigen, a reactivation of arthritis, called flare-up reaction can be induced. While acute AIA involves besides T and B cells also macrophages and immune complex formation, flare-up AIA is predominantly mediated by memory T cells [68] (Fig. 1; Tables 1 and 2). Susceptibility to AIA is not MHC class II restricted, and this makes the model useful for studies involving transgenic and gene knockout mice.

### Streptococcal Cell Wall-Induced Arthritis

A single systemic injection of group A streptococcal cell wall (SCW) peptidoglycan-polysaccharide complexes results in the development of a chronic erosive polyarthritis in genetically susceptible female Lewis rats [82]. A variation of this model involves the local injection of SCW directly into the ankle joint in rats or knee joint in mice potentially followed by systemic challenge with SCW. The initial intra-articular application of SCW causes a local, acute TLR2/NOD2-driven inflammatory reaction. This acute phase is clinically evident within 24 h after the injection, and it is histologically characterised by neutrophil and monocyte infiltration into the synovium (Fig. 1; Tables 1 and 2). The systemic (intravenous) challenge with SCW causes pronounced reactivation of the arthritic response in the joint [55]. This model of ‘reactivation’ results in an immunologically mediated inflammatory response that mimics the exacerbation of a chronic arthritic course. The chronic erosive arthritic stage is related to effector T cell activation and the dysregulation of macrophage function, characterised by accumulation of mononuclear cells with release of pro-inflammatory cytokines and erosive destruction of subchondral and peri-articular bone and cartilage [57, 83, 84]. Local challenge with SCW leads to a reactivation of arthritis and a flare-up reaction can be induced; this results in chronic inflammation, bone erosion and cartilage damage without involvement of adjuvant such as CFA.

### Spontaneous Models

#### K/BxN

K/BxN mice, which express both the T cell receptor transgene *KRN*, a TCR specific for a peptide from bovine pancreatic ribonuclease and the MHC class II molecule Ag7, develop arthritis [85]. In this model, the T cell receptor recognises the ubiquitous cytoplasmic enzyme glucose-6-phosphate isomerase (GPI) and provokes, through B cell differentiation and proliferation, high levels of anti-GPI antibodies. These antibodies are directly pathogenic upon transfer and appear to recognise endogenous cationic GPI, which seems to associate preferentially with the cartilage surface, in a complement- and FcγR-dependent manner [60, 86, 87]. This process is known as K/BxN serum transfer model.

#### SKG and gp130

Sakaguchi et al. [61] described a model of arthritis that occurred spontaneously (SKG). These mice harbour a mutation of the gene encoding the Src homology 2 domain of z-associated protein 70 (ZAP70), a key signalling molecule in T cells [61]. The aberrant T cell receptor signalling, as a result of aberrant ZAP-70, leads to a failure in thymic deletion and the emergence of auto-immune T cells.

Mice with a homozygous mutation in the gp130 IL-6 receptor subunit spontaneously develop arthritis due to enhanced gp130-mediated STAT3 activation and develop lymphocyte-mediated RA-like joint disease [62].

#### IL-1Ra^−/−^

Transgenic IL-1a overexpression was shown to induce chronic, destructive arthritis [88]. On the other hand, IL-1 receptor antagonist (IL-1Ra)-deficient mice spontaneously develop arthritis due to an increased sensitivity to IL-1. Elimination of IL-1Ra results in a model of arthritis dependent on T cells. When generated on a BALB/c (but not on C57BL/6), this mouse spontaneously develops a polyarthritis. These mice showed synovial and peri-articular inflammation, with invasion of granulation tissue and articular erosion. It also generates antibodies against type II collagen, IgG and (unlike the SKG mouse) double-stranded DNA but not IgM rheumatoid factor [63]. Disease is evident as early as 5 weeks of age, with morbidity exceeding 80 % by 8 weeks.

#### hTNF.tg

Human TNF-α transgenic (hTNF.tg) mice, which possess a modified human TNF-α gene, that resulted in pronounced TNF-α overexpression developed chronic inflammatory arthritis, from which no joints are spared, with a 100 % incidence. The histopathology in this model is characterised by hyperplasia of the synovium, inflammatory infiltrates in the joint space, pannus formation and cartilage and bone destruction. hTNF.tg mice are used as a model of RA for the inflammatory and bone destruction phases since this arthritis bypasses the adaptive initiation phase of arthritis [64].

#### BXD2

The BXD2 recombinant inbred mouse generated by inbreeding the intercrossed progeny of C57BL/6J and DBA/2J mice for more than 20 generations develop spontaneous erosive arthritis [65]. This progresses as the mice age. These mice show hallmarks of autoimmune disease, including increasing titers of circulating immune complexes and correlate with joint disease [66].

#### DNase II^−/−^IFN-IR^−/−^

A large amount of chromosomal DNA is degraded during programmed cell death and definitive erythropoiesis. DNase II is an enzyme that digests the chromosomal DNA of apoptotic cells and nuclei expelled from erythroid precursor cells after macrophages have engulfed them. DNase II^−/−^ mice die as embryos as a result of the constitutive production of interferon-β (IFN-β), and this lethality can be rescued by a deficiency of the IFN-I receptor (IFN-IR) gene. DNaseII^−/−^IFN-IR^−/−^ mice develop an inflammatory polyarthritis associated with high levels of anti-cyclic citrullinated peptide antibody and rheumatoid factor. Inadequate degradation of mammalian DNA from erythroid precursors and apoptotic cells by macrophages lead them to produce TNF-α, which activates synovial cells to produce various cytokines, leading to the development of chronic polyarthritis [67].

## Pro-inflammatory Cytokines in Bone Remodelling

### TNF-α

TNF-α is expressed mainly by macrophages and synovial lining cells, as well as by activated T cells, within the RA-inflamed joint [89]. Within the RA-inflamed joint, TNF-α is a dominant pro-inflammatory cytokine and is able to induce the production of other pro-inflammatory cytokines (IL-1, IL-6 and IL-8) [90].

TNF-α acts in synergy with RANKL and prompts robust osteoclastogenesis by osteoclast precursors, via TNF type 1 receptor (TNFr1) and deletion of TNFr1 abrogates this response [91]. TNF-α promotes the survival of differentiated mature osteoclasts [92]. On the other hand, interactions between TNF-α and IL-1 result in osteoclastic activity independently of RANKL [93].

TNF-α induces expression of the stromal cell product M-CSF, which maintains survival and longevity of osteoclast precursors. The fact that M-CSF plays a central role in TNF-induced osteoclastogenesis is confirmed by the capacity of an antibody directed against the M-CSF receptor, c-Fms, to completely arrest pathological osteoclastogenesis and bone resorption, inflammation in a serum-transfer arthritis model [94].

Osteoclast-associated receptor (OSCAR) is a key receptor in the process of osteoclast differentiation, expressed by osteoclasts at the erosion front and by mononuclear cells around synovial microvessels. Serum levels of soluble OSCAR were lower in RA patients than in healthy controls. Moreover, monocytes with high OSCAR expression exhibited an enhanced potential to differentiate into osteoclasts. TNF-α induces OSCAR expression in monocytes of RA patients, facilitating their differentiation into osteoclasts and bone resorption [95].

The relevance of TNF-α for arthritic bone destruction has been demonstrated in several experimental models and was finally confirmed by clinical trials. As discussed in ‘hTNF.tg’, hTNF.tg mice developed chronic inflammatory arthritis, with hyperplasia of the synovium, inflammatory infiltrates in the joint space, pannus formation and cartilage and bone destruction [64]. Blocking either TNF-α (using anti-TNF-α antibodies) or RANKL (using OPG.Fc) signalling in this murine model results in reduced osteoclastogenesis and bone erosion [25]. Low-dose TNF-α-inhibiting therapy, with adalimumab, also reduces bone erosions by reducing the number of circulating and joint-invading osteoclast precursors. This effect is uncoupled from its anti-inflammatory action [26]. On the other hand, treatment with exogenous TNF-α increase numbers of CD11b1 osteoclast precursor cells [96]. In CIA, the application of TNF-specific neutralising antibodies reduced disease activity and bone damage [97]. In mice with a FcγRIIB-deficient C57BL/6 background, TNF-α is indispensable while IL-17 is dispensable in the pathogenesis of arthritis. In this model, TNF-α mediates the increase in frequency of osteoclast precursors in circulation and their migration into the joints. TNF-α also decreases OPG expression, leading to up-regulated osteoclastogenesis associated with severe cartilage and bone destruction [98].

Although the observation in the murine models is largely mediated by the anti-inflammatory effects of TNF inhibition, direct reduction of osteoclast-mediated bone loss and augmentation of osteoblast-mediated bone formation are potential mechanisms by which TNF-α inhibition reduces structural damage in RA.

**Fig. 2:**
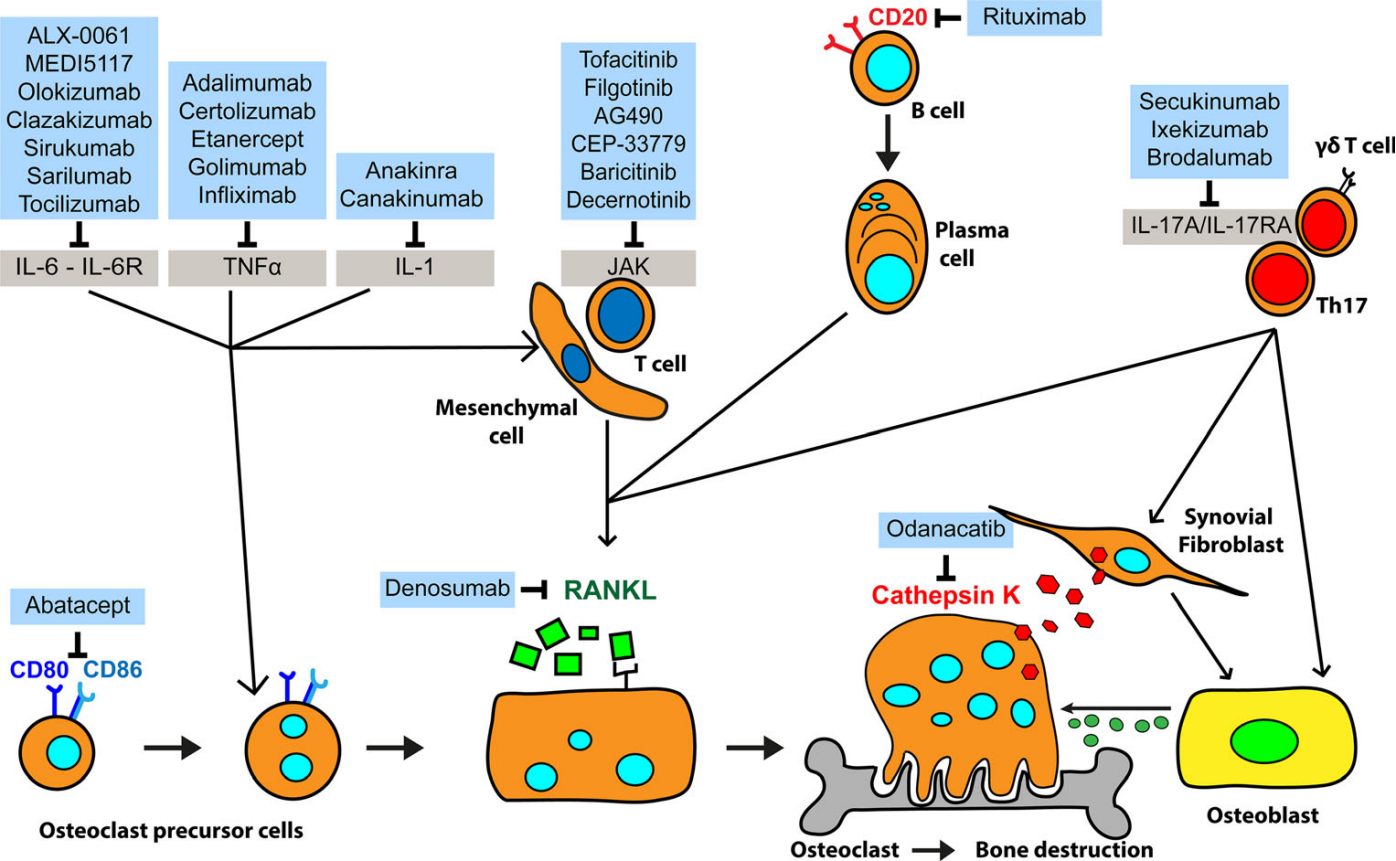
New anti-rheumatic drugs targeting osteoclastogenesis and bone loss. RANKL is a key cytokine in osteoclastogenesis and bone resorption. Downregulation of RANKL expression by mesenchymal and T cells can be achieved by targeting IL-6, TNF and IL-1. Also, depleting B cells with an antibody targeting CD20 can result in lower RANKL expression. RANKL itself can be blocked directly by denosumab. Small-molecule tyrosine kinase inhibitors can be used to block T cell activation. Abatacept can interfere with osteoclast formation by targeting CD80 and CD86. IL-17A acts on synovial fibroblast and on osteoblast, influencing osteoclast differentiation and activity. Odanacatib inhibits cathepsin K, preventing collagen and other matrix protein degradation during bone resorption

The efficacy and safety of the TNF-α antagonists infliximab, etanercept, adalimumab, golimumab and certolizumab in RA patients were demonstrated in several clinical studies, and these drugs are now frequently used in clinical practice [99] (Fig. 2).

### IL-1

The pro-inflammatory cytokine IL-1 belongs to a family of cytokines that includes IL-1α, IL-1β, IL-18 and the recently called cytokine IL-36 and IL-37. In RA joints, activated macrophages and synovial fibroblasts are sources of IL-1 production [88, 100]. IL-1 signals through its cognate receptor, IL-1R1 complexed with IL-1RAcP. IL-1 receptor antagonist (IL-1Ra) is a soluble protein that competes with IL-1 for binding to the IL-1R1 receptor [101].

IL-1 stimulates bone resorption through a primary action on osteoblasts, which are induced by IL-1 to transmit a short-range signal that stimulates osteoclastic bone resorption [102]. In vitro, IL-1 promotes the fusion of osteoclast precursors [103] and prolongs the survival of mature osteoclasts [104]. IL-1 also has an important role in osteoclast activation [105]. IL-1 is able to exacerbate osteoclastogenesis by cooperating with RANKL and M-CSF, while TNF-α is not involved in this IL-1-stimulated osteoclast differentiation pathway [106].

Data from various animal models suggest an important role for IL-1 in the pathogenesis of inflammatory arthritis. Overexpression of IL-1α or IL-1β or deficiency of the soluble IL-1Ra in arthritis models resulted in the development of disease, which is associated with bone and cartilage destruction [63, 88, 107, 108]. Mice deficient of IL-1R1 do not develop arthritis after KRN serum transfer [109].

Blocking TNF-α and IL-1 in the hTNF.tg mouse model leads to an almost-complete remission of disease, suppression of osteoclast differentiation, synovial inflammation, bone erosion and cartilage. Articular changes caused by chronic overexpression of TNF-α are not completely neutralised by monotherapies that target TNF-α, IL-1, or RANKL. Blocking TNF-α alone reduces the numbers of osteoclasts and bone erosion; however, recombinant IL-1Ra treatment is not as effective as TNF-α treatment [110].

In hTNF.tg mice that are deficient for IL-1, cartilage destruction was completely blocked and bone erosion and osteoclast formation partly reduced despite the presence of synovial inflammation. Protection of cartilage is based on the loss of IL-1 on hematopoietic cells. This study suggested that TNF-mediated cartilage damage is completely and TNF-mediated bone damage is partially dependent on IL-1, suggesting that IL-1 is a crucial mediator for inflammatory cartilage and bone degradation [111]. Moreover, lack of IL-1 completely reversed the increase osteoclast formation and bone resorption in hTNF.tg mice and the increase levels of RANKL in these mice. Structural parameters and osteoclast and osteoblast numbers were indistinguishable from wild-type mice [112].

IL-1Ra exposure or genetic ablation of IL-1RI ablated TNF-stimulated osteoclastogenesis, in vivo and in vitro. IL-1 directly targeted osteoclast precursors and promoted the osteoclast phenotype in a TNF-independent manner in the presence of permissive levels of RANKL. IL-1 is able to induce RANKL expression by stromal cells and directly stimulate osteoclast precursor differentiation. Thus, IL-1 mediates the osteoclastogenic effect of TNF by enhancing stromal cell expression of RANKL and directly by stimulating differentiation of osteoclast precursors [113].

In contrast to these in vivo and in vitro data, targeting IL-1 has not yet provided powerful therapeutics for the treatment of RA [114] (Fig. 2).

### IL-6

IL-6 belongs to the family of cytokines that includes IL-11, leukemia inhibitory factor (LIF) and oncostatin M (OSM). The signalling pathway of IL-6 includes two molecules, a specific receptor for IL-6 and a cell-surface glycoprotein called gp130 as a signal transducer [115]. IL-6 can signal via its membrane bound IL-6 receptor but also via trans-signalling, meaning that IL-6 forms a complex with soluble IL-6 receptor. This complex can bind to gp130 on the cell membrane of cells trigging IL-6 signalling. IL-6 singling is possible then on cells lacking IL-6 receptor on the membrane. IL-6 is a pro-inflammatory cytokine with pleiotropic biological activities. This cytokine is produced by a variety of cell types in the inflamed RA bone microenvironment including macrophages, fibroblast-like synoviocytes and chondrocytes [116]. IL-6 contributes to the induction and maintenance of the autoimmune process through B cell modulation and Th17 cell differentiation. In patients with RA, synovial fluid levels [117, 118] and circulating levels of IL-6 [119] are increased. Moreover, in synovial fluid IL-6 soluble receptor levels correlate with progressive joint damage in RA [120].

Several studies tried to elucidate the role of IL-6 in bone formation/resorption. Kurihara et al. demonstrated that IL-6 enhances osteoclast-like multinucleated cell formation in long-term human bone marrow cultures by inducing IL-1β release [121]. IL-6 soluble receptor triggers osteoclast-like multinucleated cell formation by IL-6, suggesting that locally or systemically produced soluble IL-6 receptor is involved in IL-6-mediated osteoclast recruitment and osteoclastic bone resorption [122]. More recently, it was demonstrated that IL-6 regulates osteoclast differentiation by modulating its interaction with the soluble IL-6 receptor complex expressed by osteoblast lineage cells, leading to an up-regulation of cyclooxygenase (COX)-2-dependent prostaglandin E2 (PGE2) synthesis. This, in turn, up-regulates RANKL expression while down-regulating OPG expression, resulting in enhanced osteoclastogenesis [36]. In vitro, blocking of IL-6 receptor reduced osteoclast formation in mouse monocyte cells stimulated with either RANKL or RANKL plus TNF-α [123]. IL-6 receptor expression increases during in vitro osteoblast differentiation and IL-6 function as a differentiation regulator of preosteoblast cells but as an apoptosis initiator in more mature cells [124].

Mice overexpressing IL-6 develop osteopenia with severe alterations in cortical and trabecular bone microarchitecture, as well as uncoupling of bone formation from resorption, with decreased osteoblast and increased osteoclast number and activity [125]. On the other hand, mice deficient in IL-6 are protected against inflammation and bone destruction in an AIA model [126, 127]. Moreover, IL-6 is an important mediator of bone destruction in AIA once it regulates T cell production of key osteoclastogenic cytokines and inflammation-induced bone marrow osteoclast differentiation [128]. Mice lacking IL-6 on the DBA/1J are completely protected from CIA, accompanied by a reduced antibody response to type II collagen and the absence of inflammatory cells and tissue damage in knee joints. However, removal of IL-6 in hTNF.tg mice does not affect the development of the disease, showing that IL-6 plays a crucial role in the development of autoimmune CIA but not in the TNF-α-dependent inflammatory arthritis (Table 2) [129]. Blocking the IL-6 receptor in a murine CIA model delayed the onset of inflammation and reduced joint destruction. In addition, administration of IL-6 receptor neutralising antibodies at the time of CIA induction completely abolished the inflammatory response indicating that IL-6 plays an important role in the initiation of arthritis [130]. Furthermore, administration of blocking antibodies against the IL-6 receptor in hTNF.tg mice did not inhibit inflammation as described by Alonzi et al. However, it significantly reduced osteoclast formation and bone erosion [123], by reducing the number of osteoclast precursors in bone marrow [131]. In several studies, performed in different arthritis mouse models, blocking IL-6 receptor was very successful. As a result of these studies, a humanised anti-IL-6 receptor monoclonal antibody, tocilizumab [132], entered into clinical trials and it has been shown to be an effective treatment in several large phase III clinical trials in RA with rapid and sustained improvement in disease activity, reducing radiographic joint damage and improving physical function [133]. Since then several agents binding either IL-6 receptor including sarilumab [134], ALX-0061 [135] or IL-6 sirukumab [136], clazakizumab [137], olokizumab [138] or MEDI5117 [139] were developed and are now in clinical trials (Fig. 2).

### IL-17

IL-17 family of cytokines consists of six members, IL-17A-IL-17F. IL-17A is not only produced by Th17 but also by other cells such as, CD8^+^ T cells, CD4^−^CD8^−^ γδ T cells, natural killer (NK) cells, innate lymphoid cells (ILC3) and potentially by mast cells and neutrophils.

The presence of IL-17-producing cells, in particular CD4^+^IL-17A^+^ cells, in blood and synovial fluid of RA patients has been correlated with inflammatory activity [140]. IL-17A levels in synovial fluids are significantly higher in RA patients compared with osteoarthritis patients [141].

Th17 cells besides IL-17A are also able to produce other cytokines such as IL-17F, IL-21, IL-22 and GM-CSF. Th17 cells can be differentiated from naïve CD4^+^ T cells by TGF-β and IL-6 or IL-21. The pro-inflammatory cytokine IL-23 is not required for the polarisation of Th17 cells but is crucial in their maturation and stability [142]. The retinoic acid-related orphan nuclear hormone receptor (ROR)γt acts as a lineage-specific transcription factor [143]. Th17 cells express the chemokine receptor CCR6. Inflamed synovial cells in both SKG arthritis and human RA also produce CCL20, the ligand for CCR6, facilitating the migration of arthritogenic Th17 cells to inflamed joints [144]. As mentioned above, Th17 cells are not the only cell type able to produce IL-17. Also, γδ T cells and potentially neutrophils are able to produce IL-17A. Equal numbers of CD4^+^ Th17 and IL-17 producing γδ T cells are found in the joints of CIA mice, and in vitro, both populations similarly induce osteoclastogenesis. However, individual depletion and adoptive transfer studies revealed that, in vivo, Th17 cells dominated with regard to bone destruction [50].

In mouse, Th17 cells directly contribute to bone damage as they express receptor activator of RANKL and have the ability to activate RANK expression on bone-resorbing osteoclasts [144]. IL-17 induces differentiation of osteoclast progenitors into mature osteoclasts in vitro, by first acting on osteoblasts, which stimulates both COX-2-dependent PGE2 synthesis and osteoclast differentiation factor gene expression [141]. Treatment of human monocytes with only IL-17 induces osteoclastogenesis. This effect could be inhibited by adding OPG and infliximab, suggesting that TNF-α and RANKL are, at least in part, responsible for the IL-17-induced osteoclastogenesis [145]. IL-17A gene transfer, using minicircle DNA, induced the expansion of IL-17RA^+^CD11b^+^Gr1^low^ osteoclast precursors. The expansion preceded noticeable joint inflammation [146]. In CIA mice overexpression of IL-17, using a viral vector, in the knee joint promotes osteoclastic bone destruction by enhancing RANKL expression and up-regulating the RANKL/OPG ratio in the synovium. Systemic OPG treatment prevents joint damage induced by local overexpression of IL-17 [147].

Interestingly, in mice lacking IL-17, CIA was markedly suppressed. In this model IL-17 is responsible for the priming of collagen-specific T cells and collagen-specific IgG2a production [148]. Lubberts et al. used anti-IL-17 antibody therapy to demonstrate that IL-17 plays a role not only in early stages of CIA but also during disease progression. Anti-IL-17 antibodies therapy suppress joint inflammation and prevent cartilage and bone destruction. Furthermore, treatment reduces IL-6 levels and the number of synovial IL-1-positive and RANKL-positive cells [149]. Neutralisation of IL-17, in a T cell-mediated model of AIA, prevents joint swelling and significantly suppress joint inflammation, cartilage proteoglycan depletion and reduces bone erosions. Synovial expression of cathepsin K, RANKL, IL-1β and TNF-α was suppressed by blocking IL-17 [59]. Transfer of IL-17-deficient donor bone marrow into CIA DBA/1J mice inhibits development and severity of clinical arthritis, due to reduction in the secretion of the pro-inflammatory cytokines TNF-α, IL-1β and IL-6 [150]. Mice lacking IL17-RA were similarly to IL-23-deficient mice completely protected against CIA [151] and from bone pathology. Mice deficient in IL-17 or IL-23 present less bone destruction and osteoclast formation in a lipopolysaccharide (LPS)-induced model of inflammatory bone destruction, which is not induced by an autoantigen but is T cell dependent. This suggests that IL-23-stimulated proliferation of Th17 cells plays a pivotal role in inflammatory bone destruction by inducing RANKL through an IL-17 effect on mesenchymal cells [152].

The development of spontaneous arthritis is completely suppressed in the progeny of IL-1Ra-deficient mice when crossed with IL-17-deficient mice, indicating that IL-17 and IL-1 are necessary for this spontaneous development of arthritis [153].

Several blockers for IL-17 pathway are been evaluated in clinical trials as secukinumab, a fully human IgG1k anti-IL-17A monoclonal antibody [154, 155], ixekizumab (a humanised IgG4 anti-IL-17A monoclonal antibody) [156, 157] or brodalumab, a fully human IgG2 anti-IL-17RA monoclonal antibodies [158, 159] (Fig. 2). However, none of these are yet approved for the treatment of RA.

### IL-23

Interleukin-23 is a heterodimeric cytokines belonging to the IL-12 family. IL-23 is a pro-inflammatory cytokine composed of two subunits, p19 and p40. The p40 subunit is shared with IL-12. IL-23 is mainly expressed by macrophages, monocytes and DCs. The IL-23 receptor (IL-23R) is composed of IL-12Rβ1 combined with a specific chain, IL-23R and is found on T cells, NK cells, macrophages and DCs (reviewed in [160]).

Several studies suggested a role of IL-23 in osteoclastogenesis. Chen et al. demonstrated that IL-23 drives osteoclast development by acting directly on myeloid precursor cells and indirectly by stimulating RANKL production in osteoblasts [161].

IL-23 induced osteoclastogenesis in cultures of human peripheral blood mononuclear cells (PBMCs) in the absence of osteoblasts or exogenous RANKL. This process was inhibited by osteoprotegerin, anti-IL-17 antibody or TNF inhibition, indicating the involvement of RANKL, IL-17 and TNF in IL-23-induced osteoclastogenesis [162]. Recently, a novel pathway involving IL-23 in myeloid cells was identified as a major mechanism for the stimulation of osteoclastogenesis in inflammatory arthritis. In human PBMCs, IL-23 induces the expression of MDL-1, a PU.1 transcriptional target during myeloid differentiation, which orchestrates osteoclast differentiation through activation of DNAX activating protein of 12 kDa and its ITAMs. IL-23-elicited osteoclastogenesis is independent of the RANKL pathway and uses a unique myeloid DNAX activating protein of 12-kDa-associated lectin-1^+^/DNAX activating protein of 12 kDa^+^ cell subset [163].

IL-23 induced RANKL expression by CD4^+^ T cells; however, the effects of IL-23 on osteoclastogenesis via T cells are less clear because both stimulatory and inhibitory effects have been described [164–166].

IL-23p19-deficient mice are protected from CIA. IL-23 gene-targeted mice do not develop clinical signs of disease and are completely resistant to the development of joint and bone pathology [58]. In vivo blockade of endogenous IL-23 activity by treatment with anti-IL-23 antibody attenuates CIA in rats by preventing both inflammation and bone destruction [162]. In mouse, administration of anti-IL-23p19 before clinical CIA onset results in a milder disease [58].

Systemic IL-23 exposure using hydrodynamic delivery of IL-23 minicircle DNA in vivo induced chronic arthritis, severe bone loss and myelopoiesis in the bone marrow and spleen, which resulted in increased osteoclast differentiation and systemic bone loss [167].

Clinical studies performed with antibodies targeting the p40 subunit of IL-12 and IL-23 (ustekinumab and briakinumab) or the p19 subunit of IL-23 (tildrakizumab and guselkumab) have been performed in patients with psoriasis, ankylosing spondylitis and psoriatic arthritis [168]. However, no studies were yet described for the application of these in the treatment of rheumatoid arthritis (Fig. 2).

## Bone Repair: New Tools, Possible Interventions and Perspectives

### Endochondral Ossification

In addition to animal models of inflammatory arthritis, there is a high interest in humanised in vivo models to study the link between the immune system and bone remodelling. Besides bone erosion, two of the most important factors common to many arthropathies are insufficient bone repair and excessive bone formation. Interestingly, both of these processes involve the process of endochondral ossification [169, 170], which is insufficient in one instance and undesirable in the other. In order to study the roles of specific cytokines on the various stages of bone erosion, insufficient bone formation and osteophyte formation new in vivo models are needed. In the field of regenerative medicine, much interest is now focused on the harnessing of this process to generate new bone and repair large bone defects, often mediated by mesenchymal stem cells (MSCs) [171–176]. This model could be harnessed to determine the effects of specific cytokines on all stages of bone formation and erosion in a controlled humanised model of bone formation. Indeed, the important role of TNF-α in endochondral bone formation using fracture healing models has already been established [177–179]. In this new model of endochondral ossification, MSCs can be chondrogenically primed in vitro in various biomaterials or as scaffold-free pellets. The early stages of condensation and chondrogenic differentiation in the presence of various cytokines can thus be assessed in vitro. Upon subcutaneous implantation of these constructs, they undergo complete endochondral ossification, becoming vascularised and forming marrow-containing bone. To date, this model has been used to demonstrate the effects of IL-1β on the various in vitro and in vivo stages of bone formation [180, 181]. We propose that the cytokines discussed in this review can also be combined in such a system to reproducibly determine their role in early chondrogenesis, bone formation, cell migration, vascularisation, marrow formation and ultimately, bone remodelling and erosion. This model represents an excellent system to analyse the specific effects of cytokines implicated in the sub-optimal repair of bone erosions in RA and the unwanted formation of osteophytes in spondyloarthritis (SpA) and osteoarthritis (OA).

### Novel Therapeutic Targets

Current treatment strategies for RA include nonsteroidal anti-inflammatory drugs (NSAIDs), corticosteroids, disease-modifying anti-rheumatic drugs (DMARDs), such as methotrexate, hydroxychloroquine, leflunomide or sulfasalazine, and biologic response modifiers (‘biologicals’). The latest have specific mechanisms of action, including inhibiting the action of individual cytokines, blocking cell-cell interactions and depleting certain cell types. The therapeutic goals of all these treatments are the clinical remission as well as structural joint protection, prevention of erosion formation, articular cartilage loss and peri-articular bone loss/osteoporosis in and around the affected joints. The suppression of inflammation and prevention of bone loss, are closely associated with each other, since as discussed in previous sections there are clear evidences linking both. Inflammatory RA is associated with reduced bone density and increased risk of fragility fractures/osteoporosis. Furthermore, suppression of inflammation halts generalised bone loss, thereby preventing increased fracture risk in RA patients [182].

In ‘Pro-inflammatory Cytokines in Bone Remodelling’, we mentioned the biologicals available that target TNF-α, IL-1, IL-6, IL-17 and IL-23 cytokine pathways. Now, we would like to discuss new treatment options that have as a target: B cells (rituximab), T cell costimulation (abatacept), JAK (tofacitinib), RANKL (denosumab) and cathepsin K (odanacatib).

#### B Cell Depletion (Rituximab)

Rituximab is a monoclonal chimeric anti-CD20 antibody that recognises a determinant expressed on intra-medullary pre-B- to B-memory stage lymphocytes, this antibody is used as a B cell depletive therapy [183] (Fig. 2).

The efficiency of rituximab was demonstrated in patients who had responded inadequately to treatment with a TNF-α antagonist in combination with background methotrexate therapy [184] and in those who had responded inadequately to methotrexate therapy [183]. Rituximab improved the signs and symptoms of RA 24 weeks after the start of the treatment. Among patients that do not respond to TNF-α antagonist therapy, progression of bone erosion and joint space narrowing on plain radiographs was slower in patients treated with combination treatment with rituximab compared with methotrexate alone in [185, 186].

In RA patients treated with rituximab, there was a decrease of RANKL expression in the synovium and a decrease in RANK-positive pre-osteoclasts [187] (Fig. 2). Moreover, rituximab treatment reduced levels of sera markers of bone resorption and bone formation. This reduction correlates with the decrease in disease activity [188].

Concerning the safety profile despite the fact that rituximab does not deplete fully mature plasma cells, repeated administration of the biologic agent frequently induces a reduction of immunoglobulins, particularly IgG, which may carry an increased risk of infection. Reactivation of occult hepatitis B infection has been reported in patients with RA treated with Rituximab. Moreover, rare occurrence of a usually fatal central nervous system demyelinating disease, progressive multifocal leukoencephalopathy has also been reported [189].

#### Inhibition of T Cell Costimulation (Abatacept)

Abatacept is a recombinant fusion protein comprising the extracellular domain of human cytotoxic T lymphocyte antigen 4 (CTLA4) and a fragment of the Fc domain of human IgG1 that selectively modulates the CD80/CD86:CD28 co-stimulatory signal required for full T cell activation. CTLA4 (CD152) is a surface protein on T lymphocytes, which negatively regulates T cell activity [190].

In rats with CIA, abatacept treatment results in reduced synovitis and osteoclasts number in the synovium [191]. In a hTNF.tg model, CTLA4 directly inhibit the formation of osteoclast through binding to CD80/86 on monocytes and thereby preventing these cells to develop to osteoclasts [192].

In patients with active RA inadequately responsive to a TNF antagonist, abatacept in combination with background DMARD therapy was more effective than background DMARD therapy alone in reducing the signs and symptoms of RA and improving physical function (Fig. 2). [193]. The combination of Abatacept and methotrexate was also more effective than methotrexate monotherapy in controlling the signs and symptoms of RA and in improving physical function among patients with RA active despite methotrexate therapy [194]. Moreover, in combination with background methotrexate therapy, abatacept slowed significantly the progression of bone erosion and joint cartilage space narrowing on plain radiographs [194, 195]. Abatacept treatment of inadequate responders to methotrexate reduces the progression of erosions in RA patients compared with patients treated with placebo [196].

The safety profile of abatacept is favourable, particularly in relation to serious (including opportunistic) infections in comparison with anti-TNF agents [197]. The most common infections reported are pneumonia, urinary tract infection and cellulitis. Antibodies to abatacept developed in ≤3 % of patients, with no association between immunogenicity and adverse events [198].

#### JAK Inhibitors (Tofacitinib)

The binding of cytokines, as for example IL-1, IL-2, IL-6, IL-8, GM-CSF and IL-10 to their receptors activates the Janus kinase (JAK)-signal transducer and activator of transcription (STAT), which further translocate to the nucleus and regulate gene expression. The JAK family comprises four members, namely JAK1-3 and tyrosine kinase 2 (TYK2) [199].

Studies performed by Changelian P. team demonstrated that administration of tofacitinib (CP-690,550), an inhibitor JAK3 and JAK1, in the two arthritis animal models CIA and adjuvant-induced arthritis, reduced the clinical and histological manifestations of joint inflammation including bone and cartilage damage [200]. Moreover, similar experiments performed by La Branche et al., using an adjuvant-induced arthritis model showed that tocacitinib decreases edema, inflammation and suppress osteoclast-mediated structural damage to arthritic joints, being this secondary to a decrease in RANKL production [201]. Tofacitinib reduces metalloproteinase and interferon-regulated gene expression in rheumatoid synovial biopsies, and clinical improvement correlates with reductions in STAT1 and STAT3 phosphorylation [202]. Others suggest a role for JAKs in the differentiation of human dendritic cells, once tofacitinib decreases CD80/CD86 expression and T cell stimulatory capability through suppression of type I IFN signalling [203].

In humans, the efficiency of tofacitinib was demonstrated in different clinical trials and is the first small molecule approved by the US Food and Drug Administration (FDA) for the treatment of RA (Fig. 2). Clinical trials demonstrated that tofacitinib has comparable efficacy with TNF-α inhibitor treatment in patients with RA under methotrexate treatment [204], and that tofacitinib in combination with methotrexate is also an effective treatment for TNF non-responders [205]. In patients who had not previously received methotrexate, tofacitinib monotherapy was superior to methotrexate in reducing signs and symptoms of RA and inhibiting the progression of structural joint damage [206]. Moreover, several studies demonstrated that tofacitinib has a sustained efficacy in the treatment of RA patients who have an inadequate response to methotrexate, etanercept, infliximab or adalimumab [69, 207, 208].

Relative to the safety profile of tofacitinib, treatment with this inhibitor has been associated with an increased risk of serious infections, infestations, malignancy and lymphoma. Frequently, patients had an increase in both low-density and high-density lipoprotein cholesterol, alanine aminotransferase, aspartate aminotransferase and serum creatinine levels and an reduction in neutrophil counts [199].

Several other JAK inhibitors are being developed with the aim to treat RA, among them some are in preclinical, as filgotinib (a JAK1/2 inhibitor, GLPG0634) [209], AG490 (a JAK2 inhibitor) [210], CEP-33779 (a JAK2 inhibitor) [211] or already in clinical development, as baricitinib (a JAK1/2 inhibitor, LY-3009104 or INCB028050) [212, 213] and decernotinib (a selective JAK3 inhibitor, VX-509) [214].

#### Anti-RANKL (Denosumab)

Denosumab (formerly known as AMG162) is a humanised IgG2 monoclonal antibody that inhibits RANKL activity and has been studied in clinical trials in patients with osteoporosis resulting in strongly suppressed bone resorption, by inhibiting the activation, proliferation and survival of osteoclasts [215].

In a study in RA patients, there was no evidence of an effect of denosumab on joint space narrowing or on disease activity. Addition of twice-yearly injections of denosumab to ongoing methotrexate treatment inhibited structural damage evident on magnetic resonance imaging, produced a sustained decrease in markers of bone turnover and resulted in increased bone mineral density, with no increase in the rates of adverse events as compared with placebo [216] (Fig. 2).

Patients receiving methotrexate for erosive RA treated in combination with denosumab were protected against erosion, and not only prevented bone loss but increased hand bone mineral density [217].

The rate of hospitalised infection among RA patients receiving denosumab concurrently with biologic agents for RA was not increased compared with those receiving zoledronate [218].

#### Cathepsin K Inhibitor (Odanacatib)

Cathepsin K is selectively expressed in osteoclasts and synovial fibroblasts and is secreted to degrade collagen and other matrix proteins during bone resorption.

Mutations in the cathepsin K gene are linked to pycnodysostosis, an autosomal recessive osteochondrodysplasia characterised by osteosclerosis, bone fragility and decreased bone turnover [219].

In RA patients, serum levels of cathepsin K were elevated and correlated with radiological destruction [220]. Moreover, rheumatoid factor positivity is associated with increased joint destruction and up-regulation of cathepsin k gene expression in the peripheral blood in patients treated with methotrexate [221].

The rationale for cathepsin K inhibition is that it will inhibit a protease and affect matrix degradation rather than osteoclast differentiation or apoptosis. This means that the number of osteoclasts and their function should not be reduced. This may allow osteoclast to osteoblast communication that contributes to maintaining bone formation, while suppressing bone resorption.

Indeed, animal studies demonstrated that cathepsin K inhibition reduces bone resorption without inhibiting bone formation [222, 223]. Deletion of cathepsin K in a hTNF.tg model results in reduced area of bone erosion although cathepsin K did not completely protect against inflammatory bone lesions [224]. In CIA mice, inhibition of cathepsin K delays the onset and reduces the disease severity, bone erosion and cartilage degradation [225].

Odanacatib is designed to avoid uptake by lysosomes (Fig. 2). The efficiency of odanacatib in osteoporosis treatment has been evaluated. Both phases 2 and 3 studies have been completed and fracture reduction has been demonstrated [226–228]. The report of the pivotal phase 3 fracture trial called The Long-Term Odanacatib Fracture Trial (LOFT), with the background and study design of fracture end point trial and baseline characteristics of its participants was recently described [226]. The results were presented last year but are still not fully published [229]. In the LOFT trial, odanacatib significantly reduced the risk of three types of osteoporotic fractures compared with placebo in the primary efficacy analysis, and also reduced the risk of the secondary endpoint of clinical vertebral fractures. The rates of adverse events overall in LOFT were generally balanced between patients taking odanacatib and placebo; however, morphea-like skin lesions and atypical femoral fractures occurred more often in the Odanacatib group than in the placebo group.

To our knowledge, there are no clinical studies about the possible application and efficacy of odanacatib for the treatment of RA; however, based in preclinical studies in animals and in the data obtained in the clinical trials for osteoporosis is reasonable to speculate about a positive outcome of the use of this inhibitor in the treatment of RA.

## Concluding Remarks

Osteoclasts have been recognised as the critical bone-resorbing cells. Studies in arthritis models have revealed the importance of many pro-inflammatory cytokines that can influence the activity of these cells directly or indirectly. The RANKL-RANK-OPG system is critical in the differentiation and activation of osteoclasts and many of the pro-inflammatory cytokines produced during persistent joint inflammation act via this pathway in the bone-resorbing process. Moreover, new data may indicate also RANKL-independent osteoclastogenesis for TNF-α and IL-23. The different animal models reflect different aspect of the pathogenesis of arthritis also in terms of cellular interactions and mediators involved in osteoclast activation and bone erosion. From these in vivo studies, the list of potential therapeutic options to treat RA inflammation including the bone-resorbing process is growing but the real proof needs to come from clinical trials. Humanised models to study bone formation and erosion might be useful to fulfil the gap between mouse and human studies. A real challenge for the future is how to repair damaged bone after controlling the inflammatory destructive process. New options via modulation of the Wnt signalling and the TGF-β/BMP pathway and perhaps the balance between IL-23, IL-17 and IL-22 might be a promising way to go; however, further research is needed on how to stimulate osteoblast activity after resolving inflammation.

## Abbreviations

AIA: Antigen-induced arthritis
BMP: Bone morphogenetic protein
CFA: Complete Freund’s adjuvant
CIA: Collagen-induced arthritis
CII: Type II collagen
COX2: Cyclooxygenase 2
CTLA4: Cytotoxic T lymphocyte antigen 4
DKK-1: Dickkopf-1
DMARDs: Disease-modifying anti-rheumatic drugs
FDA: Food and drug administration
GPI: Glucose-6-phosphate isomerase
hTNF.tg: Human TNF-α transgenic
IFA: Incomplete Freund’s adjuvant
IFN-β: Interferon β
IFN-IR: Interferon I receptor
JAK: Janus kinase
LIF: Leukemia inhibitor factor
LOFT: The long-term odanacatib facture trial
LPS: Lipopolysaccharide
M-CSF: Macrophage colony-stimulating factor-1
MSCs: Mesenchymal stem cells
NSAIDs: Non-steroidal anti-inflammatory drugs
OA: Osteoarthritis
OPG: Osteoprotegerin
OSCAR: Osteoclast-associated receptor
OSM: Oncostatin M
PBMCs: Peripheral blood mononuclear cell
PGE2: Prostaglandin E2
PsA: Psoriatic arthritis
PTH: Parathyroid hormone
RA: Rheumatoid arthritis
RANKL: Receptor activator of nuclear factor-kB ligand
RORγt: Retinoic acid-related orphan nuclear hormone receptor γt
SCW: Streptococcal cell wall
SpA: Spondyloarthritis
STAT: Signal transducer and activator of transcription
TNF: Tumour necrosis factor
TNFr1: TNF type 1 receptor
TRAP: Tartate-resistant acid phosphatase
TYK2: Tyrosine kinase 2
Wnt: Wingless
ZAP70: z-associated protein 70
